# Bridging morphology and genomics: p53, mismatch repair proteins, and β-catenin as immunohistochemistry surrogates for risk stratification in endometrial carcinoma

**DOI:** 10.5339/qmj.2026.21

**Published:** 2026-06-04

**Authors:** Mona Saeed, Zeinab El Shereef, Shahenda Saleh, Doaa Abdelhady

**Affiliations:** 1 Department of Pathology, Faculty of Medicine, Zagazig University, Zagazig, Egypt; 2 Department of Anatomic Pathology, Faculty of Medicine, Cairo University, Giza, Egypt; 3 Department of Obstetrics and Gynaecology, Faculty of Medicine, Zagazig University, Zagazig, Egypt; 4 Department of Clinical Oncology, Faculty of Medicine, Zagazig University, Zagazig, Egypt

**Keywords:** Endometrial carcinoma, molecular classification, immunohistochemistry, β-catenin, prognosis, risk stratification

## Abstract

**Background:**

Endometrial carcinoma (EC) is the most frequent gynecologic cancer in developed countries, with considerable biological heterogeneity. The Cancer Genome Atlas molecular classification provides important prognostic information, but genomic profiling is not always practical. Immunohistochemistry (IHC)-based surrogates using p53, mismatch repair (MMR) proteins, and β-catenin offer a practical alternative.

**Objective:**

This study aims to evaluate the prognostic value of an expanded IHC-based molecular panel (p53, MMR, β-catenin) for risk stratification, with a focus on identifying high-risk patients within the heterogeneous “no specific molecular profile” (NSMP) subgroup.

**Methods:**

This retrospective cohort study included 150 EC patients treated between 2018 and 2022. IHC classified cases as p53-abnormal (p53abn), MMR-deficient (MMRd), or p53-wild type (NSMP). Nuclear β-catenin (surrogate for CTNNB1 mutation) was assessed. Survival analysis used Kaplan-Meier and Cox regression.

**Results:**

Nuclear β-catenin positivity was found in 22.0% (33/150) of all cases and in 24.8% (26/105) of NSMP tumors. It was associated with lymphovascular space invasion (LVSI; 69.7% vs. 24.0%; *P* < 0.001) and nodal metastasis (24.2% vs. 8.5%; *P* = 0.02). Within the NSMP subgroup, β-catenin-positive patients had significantly worse 3-year disease-free survival (68% vs. 89%; *P* = 0.003). In multivariable analysis, β-catenin positivity was an independent predictor of recurrence (hazard ratio = 2.2 [95% CI, 1.1–4.5]; *P* = 0.02), alongside LVSI and non-endometrioid histology.

**Conclusion:**

An IHC panel incorporating p53, MMR, and β-catenin effectively stratifies EC risk. Nuclear β-catenin identifies a higher-risk subset within the prognostically heterogeneous NSMP group, supporting its integration into routine prognostic protocols to guide adjuvant therapy and surveillance, particularly in resource-limited settings.

## 1. INTRODUCTION

Endometrial carcinoma (EC) is the most frequent gynecologic cancer in developed countries and shows considerable biological heterogeneity beyond conventional histopathological subtypes.^1^ The Cancer Genome Atlas (TCGA) introduced a molecular classification that defines four prognostic groups: POLE-ultramutated (Polymerase Epsilon-ultramutated), microsatellite instability-high (mismatch repair [MMR]-deficient), copy-number high/p53-abnormal, and no specific molecular profile (NSMP). This system provides better prognostic and predictive information than histology alone.^2^

Since full genomic profiling is not always practical in routine settings, surrogate immunohistochemical (IHC) methods have been established as reliable alternatives. ProMisE-style classification, which combines p53 and MMR IHC with optional POLE testing, demonstrates strong agreement with genomic subtypes and has been adopted in several international guidelines.^3^^,^^4^ The most recent SEOM-GEICO (Spanish Society of Medical Oncology-Grupo Español de Investigación en Cáncer de Ovario) guidelines (2025) stress the clinical importance of IHC-based molecular stratification for risk assessment and individualized patient management in EC.^5^

However, the NSMP group remains clinically diverse, including tumors with both favorable and aggressive behavior. Increasing evidence shows that mutations in exon 3 of β-catenin (CTNNB1), detectable by nuclear β-catenin IHC, carry prognostic relevance. Multiple large cohort studies and systematic reviews have demonstrated that nuclear β-catenin identifies a subgroup of low-grade, early-stage endometrioid carcinomas—mainly within NSMP—with higher recurrence risk and reduced disease-free survival (DFS).^6^^,^^7^ A multicenter study published in 2024 further confirmed that nuclear β-catenin and L1CAM expression improve risk stratification, particularly in intermediate- and high-risk EC.^8^

Thus, incorporating β-catenin IHC into the standard surrogate panel with p53 and MMR is a practical, evidence-supported refinement. This expanded panel may provide more accurate risk prediction in NSMP tumors, guiding treatment choices and follow-up strategies in daily practice while bridging the gap between advanced genomic testing and routine pathology.^5^^,^^7^^,^^8^

Importantly, recent NCCN (2024) and ESGO/ESTRO/ESP (2023–2024) guidelines have also integrated molecular classification into their risk stratification and management algorithms. These updates emphasize its central role in selecting adjuvant therapy and surveillance approaches.^9^^,^^10^ Both sets of guidelines affirm that IHC-based surrogates are sufficient for most clinical decisions, especially where genomic testing is not widely available, thereby supporting the clinical use of an expanded surrogate panel including β-catenin.

Given the prognostic heterogeneity within the NSMP subgroup and the practical constraints of genomic testing in many settings, this study aimed to evaluate the prognostic impact of integrating β-catenin IHC with the standard p53/MMR panel for risk stratification in a clinical cohort of EC patients.

## 2. METHODOLOGY

### 2.1 Study design, setting, and period

This retrospective cohort study was conducted at the Departments of Pathology, Obstetrics, and Gynecology, and Clinical Oncology at Zagazig University, in collaboration with the Anatomic Pathology Department of Cairo University. The study included 150 patients diagnosed with EC between 2018 and 2022.

### 2.2 Sample size and sampling

Formalin-fixed, paraffin-embedded (FFPE) tumor samples were retrieved from institutional archives, and corresponding clinicopathologic data were collected from medical records. The sample size was determined based on the availability of complete clinical data and adequate tissue blocks meeting the inclusion criteria during the study period.

**2.2.1 Inclusion criteria**

- Patients with a histologically confirmed diagnosis of EC.
- Availability of FFPE tumor tissue blocks suitable for IHC.
- Complete clinicopathological data and surgical pathology reports.
- A minimum follow-up period of 24 months.

**2.2.2 Exclusion criteria**

- Patients with non-endometrial primary tumors (e.g., cervical, ovarian).
- Inadequate tumor tissue in the FFPE block for biomarker analysis.
- Incomplete medical records or loss to follow-up before 24 months.

### 2.3 Data collection procedure

Corresponding clinicopathologic data were collected from surgical pathology reports and electronic medical records. Hematoxylin and eosin (H&E)-stained sections were reviewed to confirm histopathological diagnosis, tumor grade, lymphovascular space invasion (LVSI), myometrial invasion depth, lymph node status, and subtype according to the WHO 2020 classification. Tumor staging followed the FIGO 2009 system.^11^

### 2.4 IHC and interpretation

IHC was performed on representative blocks using antibodies against p53 (DO-7, Dako/Agilent), MLH1 (ES05, Dako/Agilent), PMS2 (EP51, Dako/Agilent), MSH2 (FE11, Dako/Agilent), MSH6 (EP49, Dako/Agilent), and β-catenin (clone 14; BD Biosciences). Staining was visualized with the (EnVision/HRP) detection system using diaminobenzidine as the chromogen. Appropriate positive and negative controls were included.

Interpretation of IHC was performed by two independent pathologists (M.A. and S.K.), blinded to outcomes. Any discrepancies in interpretation were resolved through a joint review using a multi-head microscope to reach a consensus.

**2.4.1 Operational definitions**

- p53-abnormal (p53abn): Defined as either diffuse overexpression (>80% strong nuclear staining) or complete absence (null pattern) of nuclear staining in tumor cells. All other patterns were considered p53-wild type (p53wt).^12^^,^^13^
- MMR-deficient (MMRd): Defined as a complete loss of nuclear expression for one or more of the four MMR proteins (MLH1, PMS2, MSH2, MSH6) in tumor cells, with intact staining in internal non-neoplastic cells.^14^
- No specific molecular profile (NSMP) surrogate: Tumors exhibiting p53-wild type expression and intact MMR protein expression.
- β-catenin nuclear positivity (CTNNB1 mutant surrogate): Defined as distinct nuclear staining in ≥10% of tumor cells, regardless of concomitant membranous positivity.^15^

### 2.5 Statistical analysis

Clinicopathological data were analyzed using SPSS, version 29.0 (IBM). Categorical variables were compared using the χ² test or Fisher’s exact test, and continuous variables using Student’s t-test or Mann–Whitney U test, as appropriate. Survival outcomes included DFS, defined as the time from surgery to recurrence, and overall survival (OS), defined as the time from surgery to death or last follow-up. Kaplan–Meier survival curves were generated, and differences between groups were evaluated using the log-rank test. To determine independent prognostic factors, univariable and multivariable Cox proportional hazards regression models were applied. The variables considered for the NSMP subgroup analysis included FIGO stage, grade, LVSI, age, and β-catenin status. The full cohort model included FIGO stage, grade, LVSI, histology, p53 status, MMR status, and β-catenin status. Hazard ratios (HRs) and 95% CIs were reported. All tests were two-sided, and P-values <0.05 were considered statistically significant.

### 2.6 Ethical approval

This study was approved by the Zagazig University Institutional Review Board (ZU-IRB #1715; date: September 30, 2025). Due to the retrospective nature of the study, which involved the analysis of archived tissue and anonymized data, the requirement for informed consent was waived by the ethics committee.

## 3. RESULTS

One hundred and fifty (150) patients with histologically confirmed EC fulfilling the inclusion criteria were included. The mean age was 58.4 ± 9.2 years (range 32–81). Tumor histology was endometrioid in 128/150 (85.3%) and non-endometrioid in 22/150 (14.7%). Grade distribution was: G1–2: 122/150 (81.3%) and G3: 28/150 (18.7%). LVSI was present in 51/150 cases (34.0%; Table 1).

The cohort was predominantly endometrioid (85.3%), low-to-intermediate grade (81.3%), with LVSI present in 34.0% of cases. LVSI, lymphovascular space invasion.

Cases were categorized as follows: aberrant p53 patterns = p53-abn; diffuse overexpression (>80% strong nuclear staining); MMR protein = MMR-deficient (Table 2).

The distribution of cases according to the surrogate immunohistochemical molecular classification. The majority were classified as p53 wild-type (NSMP, 70.0%), followed by MMR-deficient (21.3%), with p53-abnormal tumors being the least frequent (8.7%).

NSMP, no specific molecular profile.

- **P53 wild-type (NSMP surrogate):** 105/150 (70.0%).
- **MMR-deficient (MMRd):** 32/150 (21.3%).
- **P53-abnormal (p53abn):** 13/150 (8.7%).

p53-abn cases were significantly enriched for high-grade (G3) and non-endometrioid histology (*P* < 0.001), greater depth of myometrial invasion (>50%; *P* < 0.001), higher LVSI rate (63.5% vs. 28.2% for P53wt), and higher frequency of positive lymph nodes (30.8% vs. 10.7% for P53wt).

Using the ≥10% nuclear staining cut-off, nuclear β-catenin (surrogate of CTNNB1 exon-3 mutation) was observed in 33/150 (22.0%) of all cases. Nuclear β-catenin was concentrated within the NSMP (P53wt) group (Table 3).

The frequency of nuclear β-catenin positivity across molecular subgroups. Overall, 22.0% of tumors were β-catenin positive. This positivity was most prevalent within the NSMP subgroup (24.8%), compared to MMR-deficient (15.6%) and p53-abnormal (15.4%) subgroups.

NSMP, no specific molecular profile.

- **NSMP (P53wt):** 26/105 (24.8%) β-catenin-positive.
- **MMRd:** 5/32 (15.6%) β-catenin-positive.
- **p53abn:** 2/13 (15.4%) β-catenin-positive.

**Figure 1.**
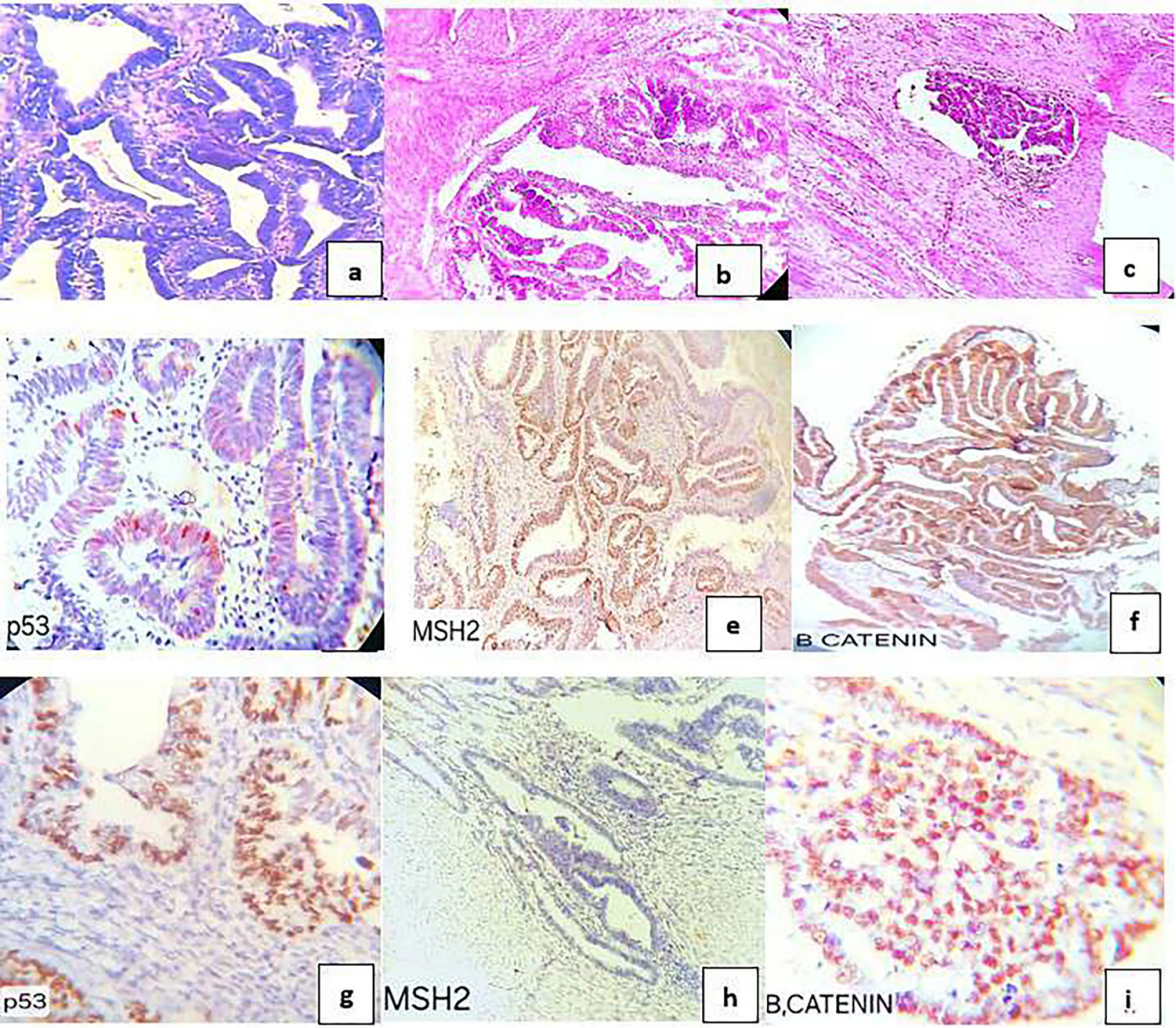
Representative immunohistochemical staining in endometrial carcinoma. (a) GI endometrial carcinoma (H&E×20). (b) GII endometrial carcinoma with myometrial invasion (H&E x20). (c) Vascular invasion by malignant acini (H&E x20). (d) p53 immunohistochemical wild-type expression showing variable, heterogeneous nuclear staining (x40). (e) MMR-proficient immunohistochemical (pMMR) tumor with preserved MSH2 nuclear staining(x20). (f) β-catenin immunohistochemical wild-type pattern with membranous staining and absent nuclear localization (x20). (g) p53 immunohistochemical -abnormal expression with strong nuclear staining (x40). (h) MMR immunohistochemical -deficient (dMMR) case showing loss of MSH2 nuclear expression (x20). (i) β-catenin immunohistochemical abnormal pattern characterized by distinct nuclear positivity in tumor cells, consistent with CTNNB1 exon 3 mutation surrogate.

Among β-catenin-positive tumors, LVSI was present in 23/33 (69.7%) vs 28/117 (24.0%) among β-catenin-negative tumors (χ² *P* < 0.001). Lymph node metastasis (pathologic): Nodal metastases occurred in 8/33 (24.2%) β-catenin-positive cases vs 10/117 (8.5%) β-catenin-negative cases (Fisher’s *P* = 0.02). Grade and myometrial invasion: β-catenin positivity was more frequent in tumors with deep myometrial invasion (>50%; *P* = 0.01) but was not restricted to high-grade cases; many positive cases were low-to-intermediate grade (Table 4; Figure 1).

The table demonstrates significant associations between β-catenin positivity and key adverse pathological features. β-catenin-positive tumors had a significantly higher prevalence of lymphovascular space invasion (69.7% vs. 24.0%; *P* < 0.001), nodal metastasis (24.2% vs. 8.5%; *P* = 0.02), and deep myometrial invasion (>50%; 63.6% vs. 37.6%; *P* = 0.01).

LVSI, lymphovascular space invasion.

**Figure 2.**
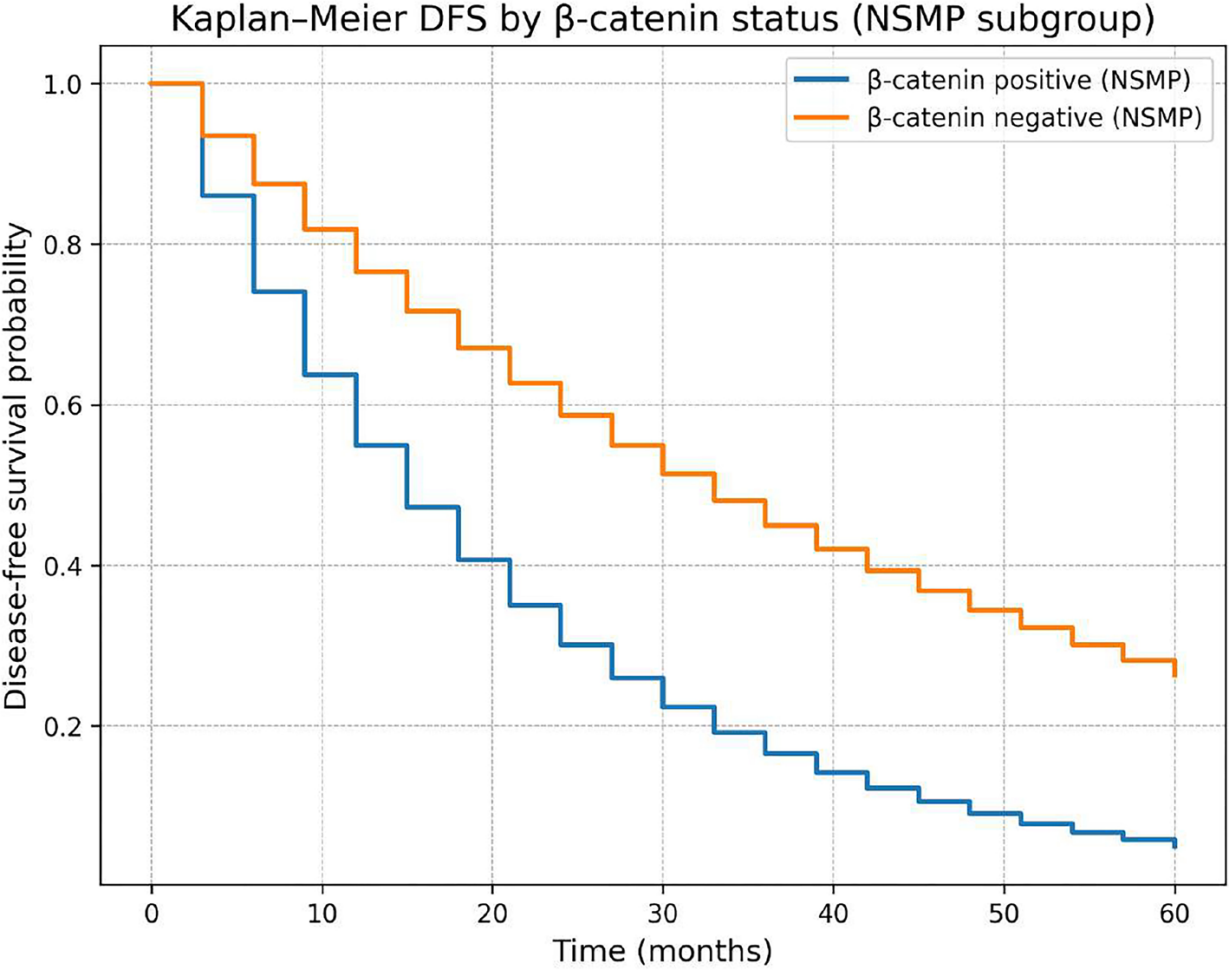
Kaplan-Meier disease-free survival (DFS) by β-catenin status in the NSMP subgroup.

The median follow-up for the cohort was 36 months (range, 6–50 months). The entire cohort 3-year DFS was 84% (95% CI 78–89%). By β-catenin status (within NSMP/P53wt group): 3-year DFS was 68% (95% CI, 52%–80%) for β-catenin-positive NSMP tumors versus 89% (95% CI, 82%–94%) for β-catenin-negative NSMP tumors. Log-rank *P* = 0.003. Univariable Cox (NSMP subgroup): β-catenin positivity HR for recurrence was 2.8 (95% CI, 1.4–5.6), *P* = 0.003. Multivariable Cox (NSMP subgroup, adjusted for FIGO stage, grade, LVSI, and age): β-catenin positivity remained an independent predictor of shorter DFS (adjusted HR = 2.2 [95% CI, 1.1–4.5]; *P* = 0.02; Figure 2).

**Figure 3.**
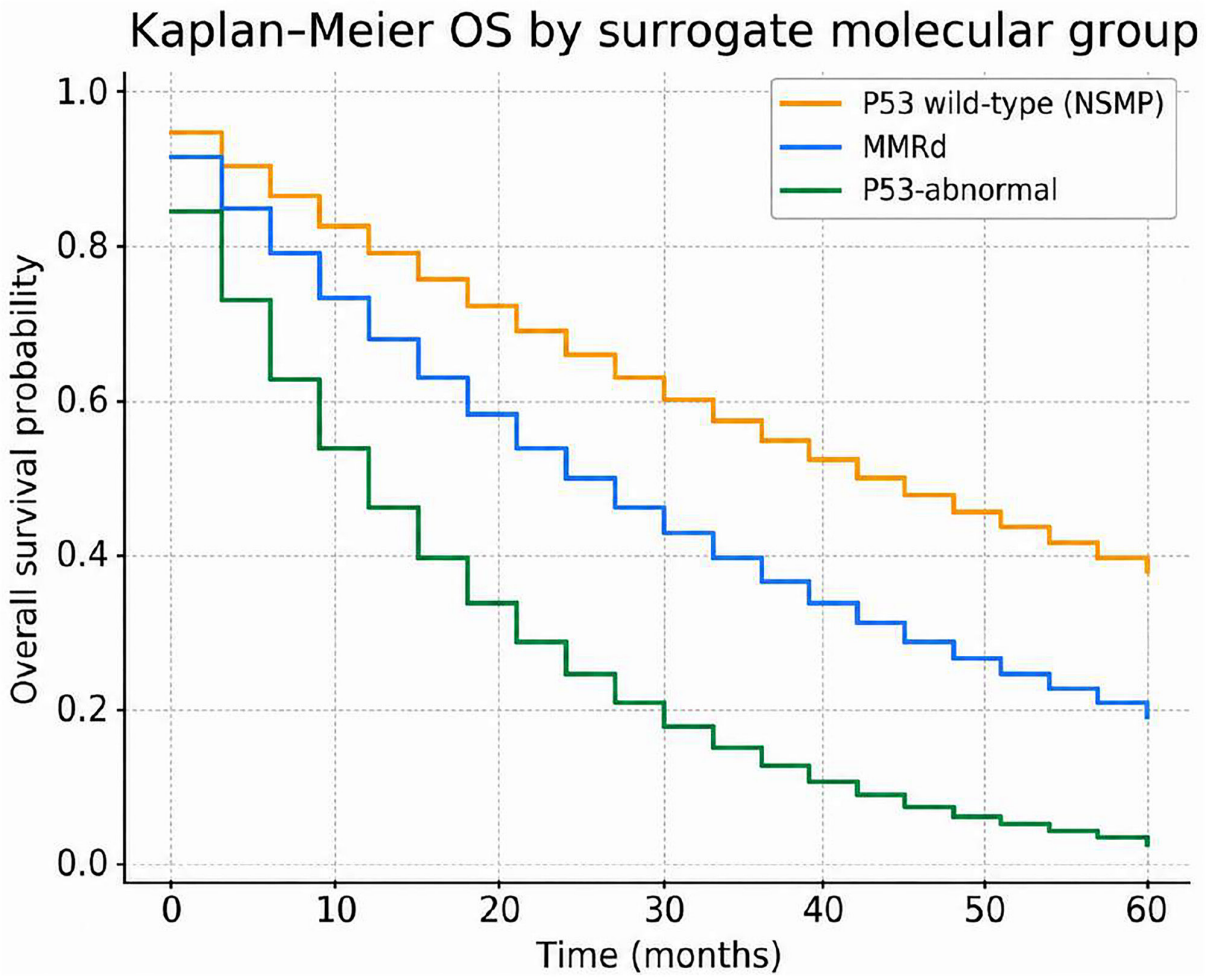
Kaplan-Meier overall survival (OS) by molecular surrogate groups (P53wt, MMRd, P53-abn).

Three-year OS of the Entire cohort was 90% (95% CI, 85–93%). By p53 subgroup: p53abn cases had the worst OS (3-year OS ~75%), MMRd intermediate (~88%), and P53wt best (~93%); Log-rank *P* < 0.05. By β-catenin (NSMP): 3-year OS was 82% for β-catenin-positive NSMP versus 92% for β-catenin-negative NSMP (log-rank *P* = 0.08). In multivariable models, β-catenin positivity showed a trend toward worse OS (adjusted HR, 1.9 [95% CI, 0.9–4.1]; *P* = 0.08) but did not reach conventional statistical significance within the available follow-up (Figure 3).

In the full-cohort multivariable model for DFS, including FIGO stage, grade, LVSI, p53 status, MMR status, and β-catenin status, independent predictors of worse DFS were: LVSI (adjusted HR, 3.6 [95% CI, 1.8–7.2]; *P* < 0.001), non-endometrioid histology (adjusted HR, 2.5 [95% CI, 1.2–5.2]; *P* = 0.01), and β-catenin nuclear positivity (adjusted HR, 2.0 [95% CI, 1.0–3.9]; *P* = 0.04). p53abn remained strongly associated with adverse features and poorer OS in models focusing on OS.

## 4. DISCUSSION

The clinicopathological profile of our cohort, with a predominance of endometrioid histology 128/150 (85.3%) and low-to-intermediate grade 122/150 (81.3%), is representative of the general EC population. This distribution is critical as it underscores the clinical challenge: within this seemingly favorable group, a subset harbors higher-risk biology. Our molecular classification successfully identified this subset, with p53abn tumors 13/150 (8.7%) showing aggressive features, thereby validating the utility of the IHC surrogate approach in our setting.

In this study of 150 EC patients, we demonstrated that classification by p53, MMR status, and β-catenin IHC provides valuable prognostic information, particularly within the NSMP subgroup. Our molecular distribution, with 70% (105/150) p53 wild-type, 21.3% (32/150) MMR-deficient, and 8.7% (13/150) p53-abnormal, closely mirrors recent large validation cohorts, where NSMP represents around 65% to 70%, MMRd approximately 20% to 25%, and p53-abn less than 10% of cases.^16^ As expected, p53-abn tumors in our series were strongly associated with adverse pathological parameters, including high-grade, non-endometrioid histology, deep myometrial invasion, LVSI, and lymph node metastasis, confirming their aggressive biological profile and poor prognosis.^16^

Nuclear β-catenin expression was observed in 22% (33/150) of cases, predominantly within the NSMP group. This prevalence is consistent with recent data showing nuclear β-catenin in 10% to 25% of endometrioid carcinomas, particularly in tumors otherwise categorized as NSMP.^16^^,^^17^ Importantly, β-catenin positivity in our study correlated with adverse features such as LVSI, nodal involvement, and deep invasion, findings that are in line with earlier studies, which demonstrated that aberrant β-catenin expression is associated with invasiveness and metastatic potential.^17^ However, a previous report^18^ found much lower rates of nuclear β-catenin positivity and no significant prognostic effect, suggesting that methodological differences in staining cut-offs, interpretation, and cohort composition may explain these discrepancies.

Our survival analyses confirm the clinical importance of β-catenin within the NSMP group. Patients with β-catenin-positive NSMP tumors had significantly worse DFS compared to β-catenin-negative cases, and β-catenin remained an independent predictor of recurrence even after adjustment for stage, grade, LVSI, and age. These findings support recent observations that CTNNB1 mutations and nuclear β-catenin staining identify a high-risk subgroup within NSMP, despite otherwise favorable clinicopathological features.^16^^,^^19^ Nevertheless, consistent with other studies, β-catenin positivity showed only a trend toward worse OS, suggesting that its most relevant impact is on recurrence risk rather than short-term mortality.^17^^,^^18^

Our findings align with studies demonstrating the adverse prognostic role of β-catenin in NSMP EC.^7^^,^^19^ However, they contrast with reports like that of Han et al. which found a very low positivity rate and no prognostic significance.^18^ This discrepancy may be attributed to differences in the studied populations (e.g., stage distribution), IHC protocols, or the chosen threshold for nuclear positivity. Our use of a ≥10% cut-off, supported by recent literature,^15^ may have enhanced the detection of clinically relevant cases.

Our multivariable models also reaffirmed LVSI and non-endometrioid histology as strong predictors of poor outcome, consistent with international guideline recommendations.^10^ The prognostic contribution of β-catenin in this context highlights its potential to refine current risk stratification, particularly within NSMP tumors, which are known to be heterogeneous in behavior. The integration of β-catenin into routine IHC panels may therefore improve the identification of patients at higher recurrence risk who might otherwise be under-treated.

The variation in reported β-catenin positivity rates across studies highlights important methodological differences, including cut-offs for nuclear staining, antibody clones, and interpretation criteria, all of which may influence reproducibility and prognostic interpretation. For example, Li et al. in a single-center study, reported nuclear β-catenin positivity in only 2.7% of endometrioid carcinomas, with no significant prognostic impact,^18^ whereas Wang et al. in a multi-institutional analysis of 576 cases, identified aberrant β-catenin expression in 10.4% of cases and found it predictive of progression-free survival in the high-intermediate risk subgroup.^7^ Follow-up duration also plays a critical role, since shorter studies may not capture enough recurrence or survival events to demonstrate significance. Furthermore, subgroup sizes, particularly β-catenin–positive NSMP cases, are often small, leading to limited statistical power and wide confidence intervals. Additional confounders such as adjuvant therapy and comorbidities may also influence outcomes, emphasizing the need for careful control in future analyses.

### 4.1 Limitations

This study has several limitations. Its retrospective design may introduce selection bias. The single-center nature and moderate sample size, particularly for subgroup analyses (e.g., β-catenin-positive NSMP, *n* = 26), limit the generalizability of the findings and statistical power. The follow-up period (median 36 months) may be insufficient to capture all late recurrences or survival events, potentially underestimating the long-term prognostic impact of biomarkers like β-catenin. Furthermore, while IHC serves as a practical surrogate, it was not validated against sequencing for CTNNB1 mutations in all cases, and the chosen cut-off for nuclear β-catenin positivity, though evidence-based, may require further standardization.

### 4.2 Recommendations and future implications

Based on the current findings, β-catenin IHC should be considered for incorporation into refined prognostic models for EC, especially within the heterogeneous NSMP subgroup. To ensure reproducibility, standardization of scoring methods is strongly recommended, with complementary validation through CTNNB1 sequencing where possible. Future research should prioritize large, prospective cohorts with extended follow-up to clarify the impact of β-catenin on OS and potential treatment benefit. Finally, exploration of Wnt/β-catenin–targeted therapies in CTNNB1-mutated tumors is encouraged, as this pathway may hold promise not only for prognostic stratification but also for personalized therapeutic strategies.

## 5. CONCLUSION

This study supports recent evidence that IHC-based molecular classification (MMRd, p53-abn, NSMP) effectively stratifies EC outcomes, with nuclear β-catenin positivity emerging as a marker of aggressive behavior within the NSMP group, reflected by lower DFS, higher LVSI, and increased nodal metastasis. While its impact on OS remains less certain, particularly in early-stage or low-grade tumors, our findings indicate that incorporating β-catenin into prognostic protocols may help identify higher-risk NSMP patients who could benefit from closer surveillance and tailored adjuvant therapy. This practical, IHC-based approach offers a cost-effective strategy for improved risk stratification, which is particularly valuable in resource-limited settings where access to advanced genomic testing is constrained.

## ACKNOWLEDGEMENTS

The authors thank the pathologists M.A. and S.K. for their expert review of immunohistochemistry slides. During the preparation of this manuscript, the authors used Grammarly for grammar and language editing. All scientific content, data analysis, interpretation, and final manuscript drafting were performed solely by the authors, who take full responsibility for the work.

## FUNDING

This research did not receive any specific grant from funding agencies in the public, commercial, or not-for-profit sectors.

## CONFLICT OF INTEREST

The author(s) declare that there is no conflict of interest.

## ETHICAL APPROVAL

This study was approved by the Zagazig University Institutional Review Board (ZU-IRB #1715; date: September 30, 2025).

## AUTHOR CONTRIBUTIONS

MS: Conceptualization, methodology, data curation, software and validation, investigation and analysis, visualization, writing–review and editing, resources, supervision, and funding acquisition. ZE: Conceptualization, methodology, investigation and analysis, visualization, writing–original draft, writing–review and editing, resources, supervision, project administration, and funding acquisition. SS: Conceptualization, methodology, writing–original draft, writing–review and editing, resources, supervision, project administration, and funding acquisition. DA: Conceptualization, methodology, data curation, software and validation, investigation and analysis, visualization, writing–original draft, writing–review and editing, project administration, and funding.

## DATA AVAILABILITY STATEMENT

The data sets generated during and/or analyzed during the current study are available from the corresponding author upon reasonable request.

## DISCLOSURE OF AI USE

The authors used an artificial intelligence–assisted tool (Grammarly) exclusively for language editing and grammar correction. The AI tool did not contribute to study design, data analysis, interpretation, or scientific conclusions. All content has been reviewed and approved by the authors, who take full responsibility for the manuscript.

## Figures and Tables

**Table 1.** Clinicopathological characteristics of the study cohort (*n* = 150).

Variable	Category	*n*	%
Age, years	Median (range)	57 (32–81)	—
Histologic type	Endometrioid	128	85.3
	Non-endometrioid	22	14.7
Tumor grade	G1–2	122	81.3
	G3	28	18.7
LVSI	Present	51	34.0
	Absent	99	66.0

**Table 2.** Distribution of molecular groups by IHC.

Molecular group	*n*	%
P53 wild-type (NSMP)	105	70.0
MMR-deficient	32	21.3

**Table 3.** β-catenin positivity by subgroup.

Subgroup	β-catenin positive *n* (%)	β-catenin negative *n* (%)
NSMP (P53wt)	26 (24.8%)	79 (75.2%)
MMRd	5 (15.6%)	27 (84.4%)
P53abn	2 (15.4%)	11 (84.6%)
Total	33 (22.0%)	117 (78.0%)

**Table 4.** β-catenin positivity versus clinicopathological features.

Feature	β-catenin positive (*n* = 33)	β-catenin negative (*n* = 117)	*P* value
LVSI present	23 (69.7%)	28 (24.0%)	<0.001
LVSI absent	10 (30.3%)	89 (76.0%)	-
Nodal metastasis present	8 (24.2%)	10 (8.5%)	0.02
Nodal metastasis absent	25 (75.8%)	107 (91.5%)	-
Deep myometrial invasion (>50%)	21 (63.6%)	44 (37.6%)	0.01
Superficial invasion (≤50%)	12 (36.4%)	73 (62.4%)	-

## References

[1] Sung H, Ferlay J, Siegel RL, Laversanne M, Soerjomataram I, Jemal A (2020). Global cancer statistics 2020: GLOBOCAN estimates of incidence and mortality worldwide for 36 cancers in 185 countries. CA Cancer J Clin.

[2] Kandoth C, Schultz N, Cherniack AD, Akbani R, Liu Y, Cancer Genome Atlas Research Network (2013). Integrated genomic characterization of endometrial carcinoma. Nature.

[3] Kommoss S, McConechy MK, Kommoss F, Leung S, Bunz A, Magrill J (2018). Final validation of the ProMisE molecular classifier for endometrial carcinoma in a large population-based case series. Ann Oncol.

[4] Fer reira EO, Schefter AM, Brustad A, Klein ME, Khalifa MA, Winterhoff B (2024). Implementation of endometrial cancer molecular subtyping into a hybrid community-academic practice. Am J Clin Pathol.

[5] Oak nin A, Gómez-Hidalgo NR, Gallego A, De Juan Ferré A, Madrid LF, Martínez AG (2025). SEOM-GEICO clinical guidelines on endometrial cancer (2025). Clin Transl Oncol.

[6] Travaglino A, Raffone A, Raimondo D, Reppuccia S, Ruggiero A, Arena A (2022). Prognostic significance of CTNNB1 mutation in early stage endometrial carcinoma: a systematic review and meta-analysis. Arch Gynecol Obstet.

[7] Wang X, Aziz AUR, Wang D,  Wang Y, Liu M, Yu X (2024). Prognostic factors and survival outcomes of immunohistochemically detection based-molecular subtypes of endometrial cancer-analysis of 576 clinical cases. Diagn Pathol.

[8] Yoon H, Suh DH, Kim K, No JH, Kim YB, Kim H (2024). Evaluation of prognostic potential of β-catenin and L1CAM expression according to endometrial cancer risk group. Gynecol Oncol.

[9] National Comprehensive Cancer Network (NCCN) Clinical Practice Guidelines in Oncology: Uterine Neoplasms. Plymouth Meeting, PA: NCCN.

[10] Concin N, Matias-Guiu X, Vergote I, Cibula D, Mirza MR, Marnitz S (2021). ESGO/ESTRO/ESP guidelines for the management of patients with endometrial carcinoma. Int J Gynecol Cancer.

[11] Pec orelli S (2009). Revised FIGO staging for carcinoma of the vulva, cervix, and endometrium. Int J Gynaecol Obstet.

[12] Vermij L, Nout R, Osse EM, Powell ME, Edmondson RJ, Genestie C (2022). p53 immunohistochemistry in endometrial cancer: clinical and molecular correlates in the PORTEC-3 trial. Mod Pathol.

[13] Wang J, Cai Y, Wang J, Fang J, Pang J, Zhang H (2025). Prevalence of atypical and subclonal p53 immunohistochemistry expression in mismatch repair deficient and/or POLE-mutant endometrial carcinomas with TP53 mutation. Lab Invest.

[14] Galant N, Krawczyk P, Monist M, Obara A, Gajek Ł, Grenda A (2024). Molecular classification of endometrial cancer and its impact on therapy selection. Int J Mol Sci.

[15] Parrish ML, Osborne-Frazier ML, Broaddus RR, Gladden AB (2025). Differential localization of β-catenin protein in CTNNB1 mutant endometrial cancers results in distinct transcriptional profiles. Mod Pathol.

[16] Vermij L, Colas E, Huvila J (2024). International validation of a computer-assisted tumour budding assessment tool in endometrial carcinoma. Diagn Pathol.

[17] Manule Y, Miskad UA, Masadah R, Nelwan B, Cangara MH, Mardiati M (2023). Prognostic value of β-catenin and L1CAM expressions in type I endometrial carcinoma. Asian Pac J Cancer Prev.

[18] Li A, Zhang M, Wang J (2024). Clinicopathological significance and prognostic value of βcatenin expression in endometrioid endometrial carcinoma. Oncol Lett.

[19] Travaglino A, Raffone A, Raimondo D, Reppuccia S, Ruggiero A, Arena A (2022). Prognostic significance of CTNNB1 mutation in early stage endometrial carcinoma: a systematic review and meta-analysis. Arch Gynecol Obstet.

